# Usefulness of peroral cholangioscopy in the differential diagnosis of IgG4-related sclerosing cholangitis and extrahepatic cholangiocarcinoma: a single-center retrospective study

**DOI:** 10.1186/s12876-020-01429-2

**Published:** 2020-08-24

**Authors:** Yasutaka Ishii, Masahiro Serikawa, Tomofumi Tsuboi, Ryota Kawamura, Ken Tsushima, Shinya Nakamura, Tetsuro Hirano, Ayami Fukiage, Juri Ikemoto, Yusuke Kiyoshita, Sho Saeki, Yosuke Tamura, Kazuaki Chayama

**Affiliations:** Department of Gastroenterology and Metabolism, Graduate School of Biomedical & Health Sciences, Hiroshima University, 1-2-3 Kasumi, Minami-ku, Hiroshima, 734-8551 Japan

**Keywords:** IgG4-related sclerosing cholangitis, Extrahepatic cholangiocarcinoma, Peroral cholangioscopy, Differential diagnosis

## Abstract

**Background:**

In the diagnosis of IgG4-related sclerosing cholangitis (IgG4-SC), differentiation from extrahepatic cholangiocarcinoma (ECC) is extremely important but is still a clinical challenge. This study aimed to elucidate the usefulness of peroral cholangioscopy (POCS) for the differential diagnosis between IgG4-SC and ECC.

**Methods:**

POCS findings for bile duct stricture were retrospectively evaluated in 17 patients with IgG4-SC diagnosed at the Hiroshima University Hospital and 53 patients with surgically resected infiltrating ECC. Mucosal surface, dilated vessels (tortuosity, caliber alteration, and disruption), and easily bleeding were compared between the groups.

**Results:**

The stricture sites of IgG4-SC evaluated by POCS were 10 extrapancreatic bile ducts and 9 intrapancreatic bile ducts. In patients with IgG4-SC, smooth mucosal surface was observed in 89% (17/19), dilated vessels in 58% (11/19) [tortuosity 82% (9/11), caliber alteration 18% (2/11), and disruption 9% (1/11)], and easily bleeding in 0%. Irregular mucosal surface and easily bleeding were observed significantly more frequently in ECC (both *P* <  0.001). The frequency of caliber alteration and disruption of dilated vessels was significantly less in IgG4-SC (*P* <  0.001 and 0.005, respectively). The sensitivity and specificity of POCS in the diagnosis of ECC were 96 and 89%, respectively. Dilated vessels in IgG4-SC were observed significantly more frequently in the extrapancreatic bile duct, especially the hilar bile duct (*P* = 0.006). Concerning image evaluation, the interobserver agreement was κ = 0.719, and the intraobserver agreement was κ = 0.768 and 0.754.

**Conclusions:**

Characteristic POCS findings of the stricture sites in IgG4-SC were smooth mucosal surface, dilated vessels without caliber alteration and disruption, and lack of easily bleeding. These POCS findings are extremely useful for distinguishing between IgG4-SC and ECC.

## Background

IgG4-related sclerosing cholangitis (IgG4-SC) is characterized by elevation of serum IgG4 levels, and dense infiltration of IgG4-positive plasma cells with extensive fibrosis in the bile duct wall. Similar to autoimmune pancreatitis (AIP), IgG4-SC is recognized as a biliary manifestation of a systemic disorder termed IgG4-related disease [1]. Since it responds well to steroid therapy, the autoimmune mechanism is presumed as the etiology. The clinical diagnostic criteria were proposed in Japan in 2012 [2], and IgG4-SC is diagnosed by combining diffuse or segmental stricture of the intrahepatic and/or extrahepatic bile duct associated with the thickening of bile duct wall, elevated serum IgG4 levels, coexistence of IgG4-related diseases (AIP, sclerosing dacryoadenitis/sialadenitis, and retroperitoneal fibrosis), and characteristic histopathological findings. The effectiveness of steroid therapy as an option is also taken into consideration. Furthermore, in the diagnosis of IgG4-SC, it is extremely important to distinguish from malignancies such as extrahepatic cholangiocarcinoma (ECC) and pancreatic cancer, and primary sclerosing cholangitis.

IgG4-SC is classified into four types based on the site of strictures due to cholangiography and diseases to be distinguished [3]. Of the four types, type 1 IgG4-SC requires differentiation from distal cholangiocarcinoma, and type 3 and type 4 IgG4-SC require differentiation from perihilar cholangiocarcinoma. The differential diagnosis between IgG4-SC and ECC is often difficult only by cholangiography [4], so comprehensive diagnosis using several factors, such as serum IgG4 level, presence or absence of other IgG4-related diseases, and pathological diagnosis of bile duct stricture is crucial. However, differential diagnosis of IgG4-SC and ECC remains an important clinical challenge.

Peroral cholangioscopy (POCS) is an examination method that allows direct observation of bile duct mucosa by inserting a small diameter cholangioscope transpapillary into the bile duct. POCS is useful for the differential diagnosis between benign and malignant bile duct stricture [5–10], and the diagnosis of the extent of superficial spread of cholangiocarcinoma [11, 12]. Inflammation of IgG4-SC is found transmurally, but the main focus of inflammation is under the epithelium of the bile duct, and the epithelium is often normal [13]. Therefore, the POCS findings of IgG4-SC may be different from those of epithelial tumor, cholangiocarcinoma, and POCS may be useful for differentiating them.

The aim of this study was to clarify the characteristic POCS findings of IgG4-SC and the usefulness of POCS in differentiation between IgG4-SC and ECC by evaluating the findings in detail.

## Methods

### Patients

A total of 70 patients, including 17 patients with IgG4-SC and 53 patients with surgically resected infiltrating ECC who underwent POCS at Hiroshima University Hospital between April 2008 and December 2017, were enrolled in this study, and POCS findings were retrospectively examined. The infiltrating ECC consisted of macroscopically nodular type and flat infiltrating type [14]. IgG4-SC was diagnosed based on the clinical diagnostic criteria of IgG4-SC [2]. POCS was performed to differentiate between benign and malignant bile duct stricture or to diagnose the extent of superficial spread of ECC. The time of POCS performed in patients with IgG4-SC was at initial diagnosis in 11 patients and at relapse in 6 patients.

Written informed consent was obtained from all patients and their families before endoscopic retrograde cholangiopancreatography (ERCP) and POCS were performed. This study was conducted in accordance with the Declaration of Helsinki and was approved by the ethics committee of Hiroshima University Hospital (approval No. E-1564).

### ERCP and POCS procedure

All POCS was performed by using a conventional dual-operator mother-baby technique. One of the two video cholangioscopes (CHF-B260 and CHF-BP260; outer diameter of 3.4 mm and 2.6 mm; working channel diameter of 1.2 mm and 0.5 mm, respectively; Olympus Medical Systems, Tokyo, Japan) was used as a baby scope. A therapeutic duodenoscope (TJF-240 or TJF-260 V; working channel diameter 4.2 mm; Olympus Medical Systems) was used as a mother scope.

First, ERCP was performed, and endoscopic retrograde naso-biliary drainage (ENBD) was performed regardless of the presence or absence of jaundice and acute cholangitis. Detailed evaluation of the bile duct was performed by cholangiography via ENBD catheter. In patients with jaundice or acute cholangitis, POCS was performed after they improved. In contrast, in patients without jaundice or acute cholangitis, POCS was performed 2 to 4 days after the initial ERCP. Immediately before POCS was performed, a saline solution was injected into the bile duct via ENBD catheter to thoroughly irrigate the inside of the bile duct. To avoid artifacts of the lesion by guidewire manipulation, the mother scope was inserted while passing the ENBD catheter through the working channel, and the a 0.025-in. guidewire was placed. After the small endoscopic sphincterotomy or papillary balloon dilatation using a balloon catheter with 6 mm diameter was performed, the cholangioscope was inserted into the bile duct with passage over the guidewire. The bile duct lumen was observed while irrigating with the saline solution through the working channel of the cholangioscope. Conventional white light endoscopy was mainly used for observation, and narrow band imaging (NBI) was used as necessary. All POCS procedures were performed under conscious sedation of the patient with intravenous administration of pentazocine and midazolam by two experienced endoscopists. Following POCS, biopsies from the stricture were performed under fluoroscopic guidance using a conventional biopsy forceps (Radial Jaw; Boston Scientific Corp., Natick, MA, USA), if necessary. After performing POCS, ENBD and intravenous administration of an antibiotic was carried out in all patients for the purpose of preventing acute cholangitis.

All cholangioscopic images were stored as both video and still images and were evaluated by two endoscopists with over 15 years of experience (Y.I. and M.S.). Based on previous reports [5–10], the POCS findings of the stricture were evaluated in the following points: (1) properties of mucosal surface: smooth or irregular (papillogranular); (2) vessels: existence of dilated vessels and its morphology (tortuosity, abrupt caliber alteration, disruption); and (3) easily bleeding. Easily bleeding was defined as spontaneous bleeding without contact of the cholangioscope or manipulation of the guidewire. The characteristics of the vessels were defined as follows with reference to the report of Kaise et al. [15]. which examined the characteristics of tumor vessels of superficial depressed gastric carcinoma by magnifying endoscopy with NBI:

1. Dilatation: presence of vessels whose calibers are twice or more than twice the calibers of surrounding reference vessels.
2. Tortuosity: presence of vessels which are unpredictably twisted or bent.
3. Abrupt caliber alteration: presence of vessels whose calibers abruptly become less than one half or more double the original size.
4. Disruption: presence of vessels which have a dead end.

### Inter- and intraobserver agreement

In an independent substudy, the inter- and intraobserver agreements of POCS images for differentiating between IgG4-SC and infiltrating ECC were assessed by an endoscopist with 11 years of ERCP experience (T.T.) and an endoscopist with 8 years of ERCP experience (N.S.). All 72 stricture sites were evaluated. Several white light images and NBI images of each stricture site were selected. All selected images were randomly arranged for pattern assessment by the two readers who were blinded to the final diagnosis of the lesion. Strictures were evaluated based on the characteristic POCS findings of IgG4-SC and infiltrating ECC in this study. Two readers diagnosed the image of one pattern in 1 day, and diagnosed another pattern 2 weeks later.

### Statistical analysis

Statistical analyses were performed using JMP Pro 12.2.0 (SAS Institute Inc. Cary, North Carolina, USA). The Chi-square test or Fisher’s exact test were used for the comparison of proportions. *P* values < 0.05 were considered statistically significant.

## Results

### Patients characteristics

The clinical profiles of all 17 patients with IgG4-SC who underwent POCS are shown in Table 1. The median age was 63 years (range, 51–77 years), and 16 patients were men and 1 patient was woman. The median serum IgG4 level was 264 mg/dl (range, 21–2590 mg/dl), and 15 patients (88%) showed an elevated serum IgG4 level (≥135 mg/dl) satisfying the diagnostic criteria. The cholangiographic classifications were 7 patients of type 1, 2 patients of type 3, and 8 patients of type 4. Coexistence of AIP was found in 15 patients (88%), and the range of pancreatic enlargement was diffuse in 5 patients, segmental in 3 patients, and focal in 7 patients. Forceps biopsy of the stricture was performed 14 patients (82%) and immunostaining of IgG4 was performed in 7 patients in which bile duct stroma with inflammatory cell infiltration was collected. However, there were no patients in which infiltration of abundant IgG4-positive plasma cells > 10 per high power field was observed, and none of the patients met the histopathological criteria of IgG4-SC [2]. Oral prednisolone was introduced or increased in 14 patients (82%). The details of 3 patients in whom steroid therapy was not introduced were as follows: 1 patient underwent spontaneous remission, 1 patient underwent surgery for the diagnosis of pancreatic cancer, and 1 patient had a history of side effect of prednisolone.

**Table 1:** Clinical profiles of the 17 patients with IgG4-SC

Characteristics	Values
Age (years)	63 (51–77)
Sex (male: female)	16: 1
Serological findings	
Serum IgG4 (mg/dL)	264 (21–2590)
Elevated serum IgG4 level (≥135 mg/dL), n (%)	15 (88%)
Serum IgG (mg/dL)	1543 (807–5530)
Total bilirubin (mg/dL)	1.2 (0.6–22.4)
ALP (IU/L)	677 (107–1331)
CA19–9 (U/L)	10 (0–107)
Cholangiographic classification, n (%)	
Type 1	7 (41%)
Type 2	0 (0%)
Type 3	2 (12%)
Type 4	8 (47%)
Other organ involvement, n (%)	15 (88%)
Autoimmune pancreatitis	15 (88%)
Sclerosing dacryoadenitis & sialadenitis	3 (18%)
Retroperitoneal fibrosis	1 (6%)
Diagnosis, n (%)	
Definite	15 (88%)
Probable	1 (6%)
Possible	1 (6%)
Steroid therapy, n (%)	14 (82%)

Data are expressed as number (percentage) or median (range)

IgG4-SC, IgG4-related sclerosing cholangitis; ALP, alkaline phosphatase; CA19–9, carbohydrate antigen 19–9

The patients with ECC were 37 men and 16 women, with a median age of 71 years (range, 42–82 years). The locations of tumor were perihilar bile duct in 30 patients and distal bile duct in 23 patients. The preoperative diagnosis of cholangiocarcinoma was obtained pathologically in 40 patients (75%), and the sensitivity of bile juice cytology, brushing cytology, and forceps biopsy were 43% (22/51), 48% (24/50), and 63% (32/51), respectively. The surgical procedures were pancreatoduodenectomy in 22 patients, right hepatectomy in 14 patients, left hepatectomy in 3 patients, extended right hepatectomy in 5 patients, hepatopancreatoduodenectomy in 5 patients, and bile duct resection in 4 patients.

Regarding POCS-related complications, acute cholangitis was seen in two patients of ECC, but it improved rapidly by conservative therapy. There were no other complications, such as acute pancreatitis and liver abscess.

### Cholangioscopic findings of IgG4-SC and ECC

**Fig. 1:**
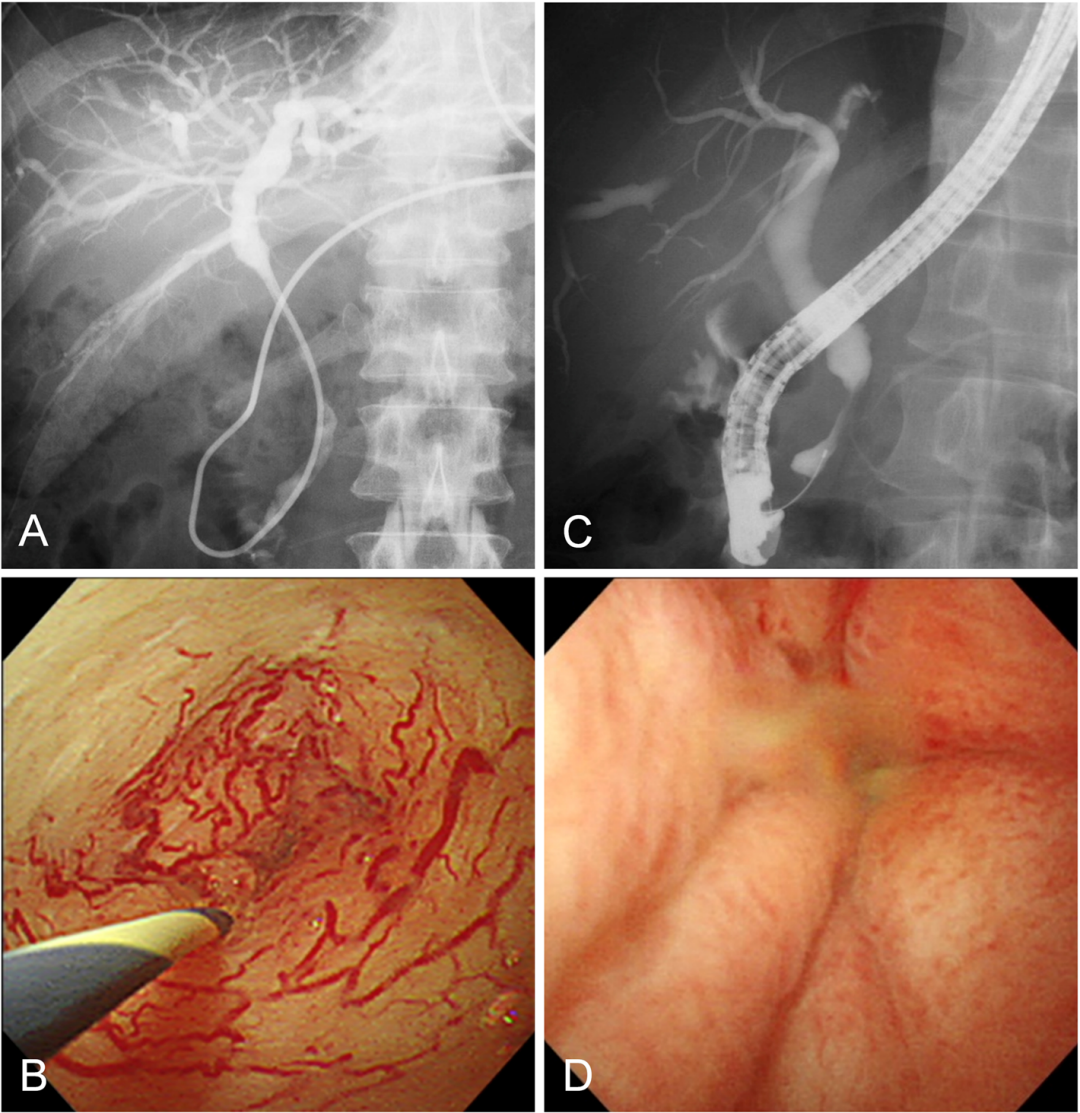
Cholangioscopic images of IgG4-related sclerosing cholangitis. **a** Stricture in the middle and hilar bile duct. **b** Dilated and tortuous vessels spreading around the stricture with smooth mucosal surface. **c** Stricture in the intrapancreatic bile duct. **d** Stricture with edematous and smooth mucosal surface

**Fig. 2:**
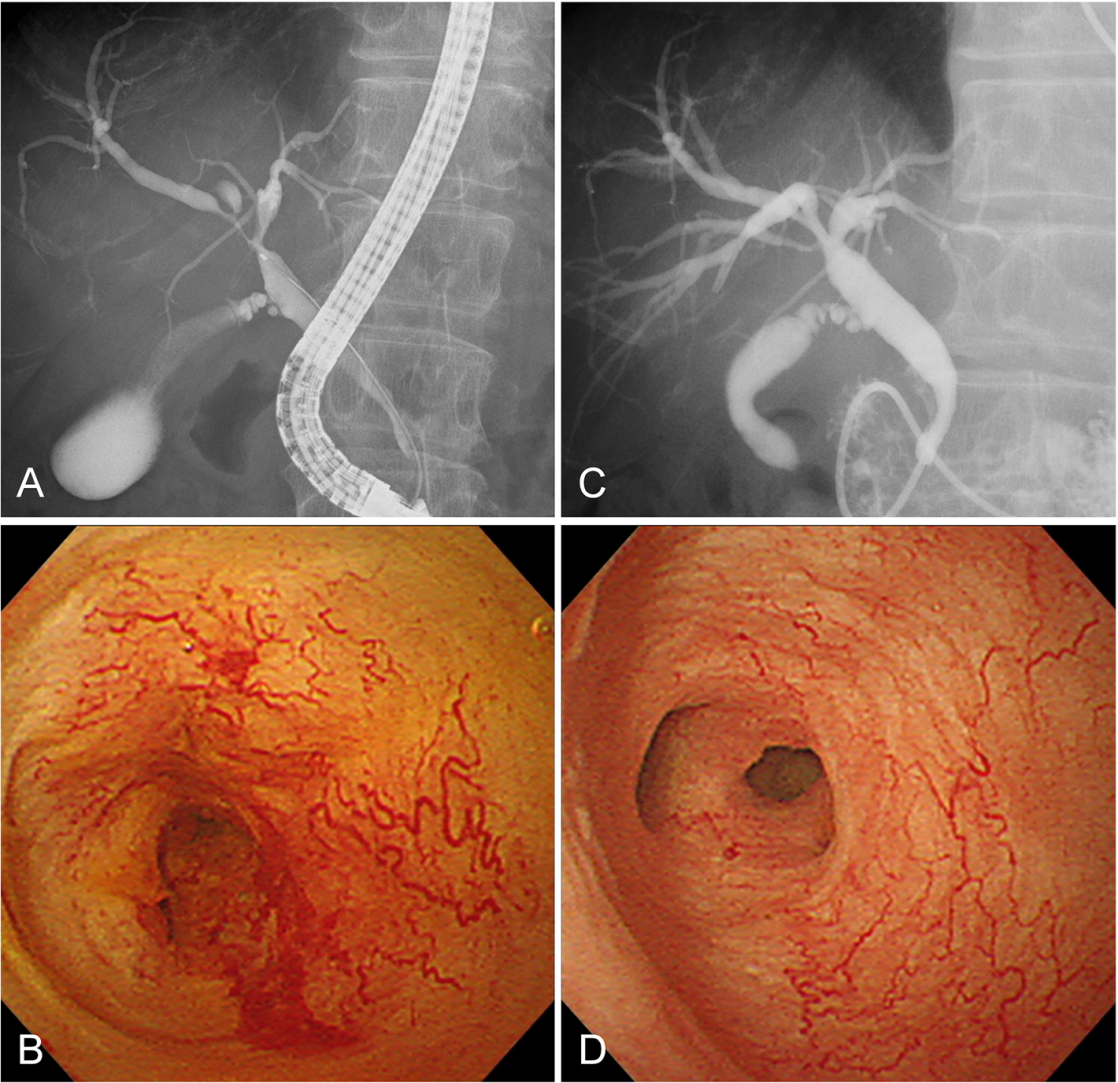
Changes in cholangioscopic images by steroid therapy in a patient with IgG4-related sclerosing cholangitis. Before steroid therapy, cholangiography showed stricture in the hilar bile duct (**a**), and dilated and tortuous vessels around the stricture (**b**). Seven days after induction of steroid therapy, stricture (**c**) and dilatation of the vessels (**d**) were markedly improved

POCS findings were evaluated at 19 sites of bile duct stricture in 17 patients with IgG4-SC (Fig. 1). The mucosal surface was smooth in 89% (17/19) of strictures. Dilated vessels were observed in 58% (11/19) of strictures. The tortuosity of the dilated vessels was observed at 82% (9/11), but the network structure was maintained, and the abrupt caliber alteration and the disruption were found at only 18% (2/11) and 9% (1/11), respectively. There was no patient showing easily bleeding. In nine patients with IgG4-SC, the stricture was re-evaluated by POCS after introduction or increase of steroid [median 9 days (range, 7–39 days)]. Improvement of stricture was confirmed in all nine patients, and improvement of vessel dilatation was confirmed in all six patients where dilated vessels were in the stricture (Fig. 2).

**Fig. 3:**
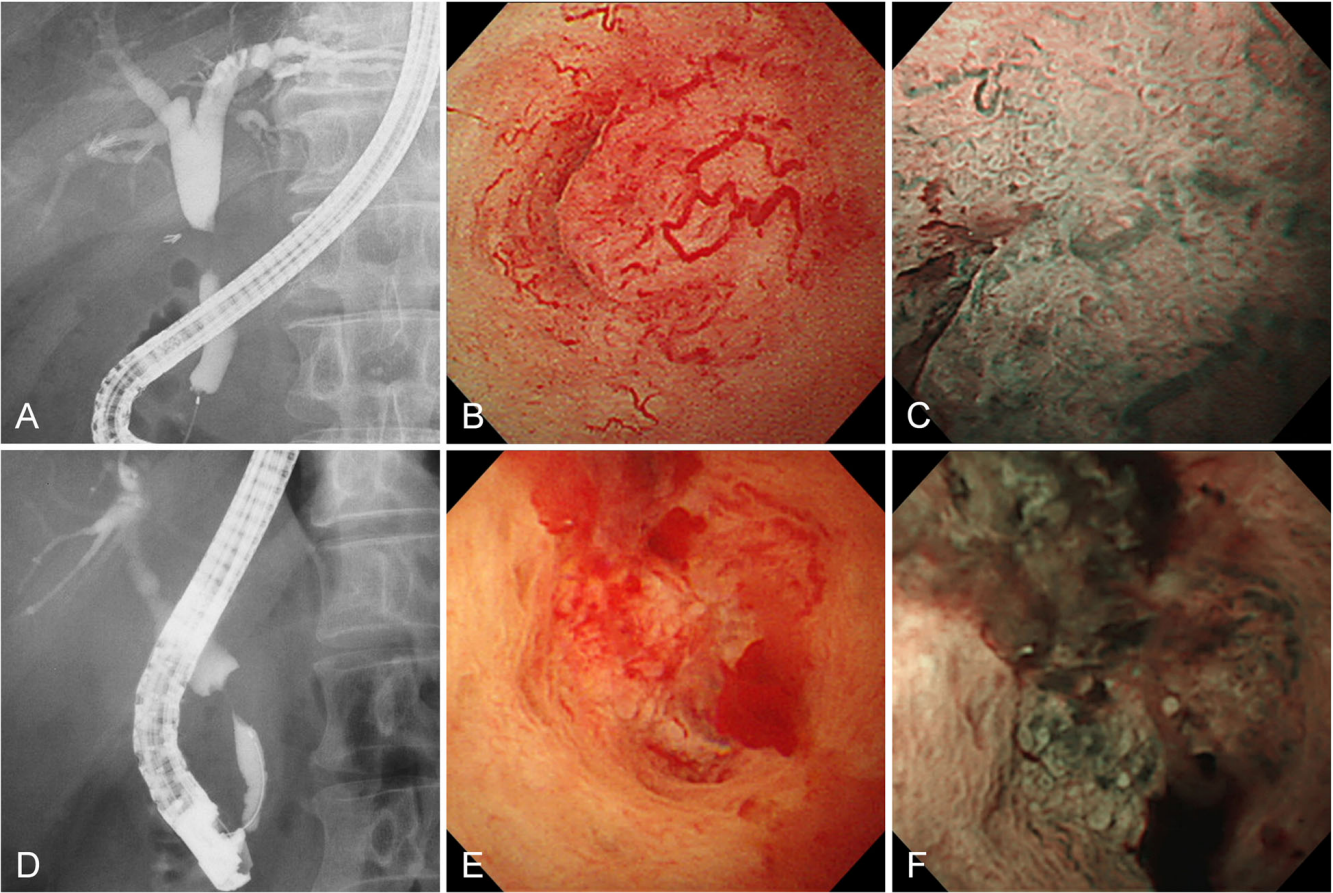
Cholangioscopic images of infiltrating type extrahepatic cholangiocarcinoma. **a** Stricture in the middle bile duct. **b** Dilated and tortuous vessels with caliber alteration and disruption. **c** Granular mucosa in narrow band imaging. **d** Stricture in the intrapancreatic bile duct. **e** Dilated and tortuous vessels with caliber alteration and spontaneous bleeding. **f** Granular mucosa in narrow band imaging.

On the other hand, the mucosal surface of ECC was irregular in 91% (48/53) of the patients (Fig. 3). Dilated vessels were found in 79% (42/53) of patients with ECC. Of the 42 ECC patients with dilated vessels, the tortuosity, abrupt caliber alteration, and disruption of the dilated vessels were found in 35 (83%), 36 (86%), and 25 (60%) patients, respectively. Easily bleeding was observed in 26 patients (49%).

A comparison of POCS findings between IgG4-SC and ECC is shown in Table 2. There was a significant difference in the property of mucosal surface and presence of easily bleeding (both *P* <  0.001). There was no significant difference in the frequency of dilated vessels (*P* = 0.070). Regarding the morphology of dilated vessels, there was no difference in the presence of tortuosity, but abrupt caliber alteration and disruption were found significantly more frequently in patients with ECC than patients with IgG4-SC (*P* <  0.001 and 0.005, respectively). When the POCS findings of malignant stricture were defined as irregular mucosal surface, presence of dilated vessels with abrupt caliber alteration and disruption, or easily bleeding, the diagnosis of ECC was 96% (51/53) for sensitivity and 89% (17/19) for specificity.

**Table 2:** Comparison of POCS findings between IgG4-SC and ECC

	IgG4-SC	ECC	
**POCS findings**	**(*n*** **= 19)**	**(*n*** **= 53)**	***P*** **value**
Mucosal surface (smooth: irregular)	17: 2	9: 44	< 0.001
Dilated vessels (present: absent)	11: 8	42: 11	0.070
Tortuosity (present: absent)	9: 2	35: 7	1.000
Abrupt caliber alteration (present: absent)	2: 9	36: 6	< 0.001
Disruption (present: absent)	1: 10	25: 17	0.005
Easily bleeding (present: absent)	0: 19	26: 27	< 0.001

*POCS* peroral cholangioscopy; *IgG4-SC* IgG4-related sclerosing cholangitis; *ECC* extrahepatic cholangiocarcinoma

### Relationship between POCS findings and site of stricture

POCS findings, site of stricture, and site of AIP lesions in patients with IgG4-SC are shown in Table 3, and a comparison of POCS findings based on the stricture site is shown in Table 4. Dilated vessels were found in 90% (9/10) of extrapancreatic bile duct strictures, whereas only 22% (2/9) of intrapancreatic bile duct strictures. Therefore, dilated vessels were found significantly more frequently in extrapancreatic bile duct strictures, particularly the hilar bile duct, than in intrapancreatic bile duct strictures (*P* = 0.006). There was no significant relationship between the property of mucosal surface and the site of stricture, but the mucosal surface of intrapancreatic bile duct stricture was smooth in all patients. In terms of the relationship between the site of stricture and the site of AIP lesion, intrapancreatic bile duct stricture was accompanied by enlargement of the pancreatic head in all nine patients. In contrast, there was no significant difference in POCS findings between perihilar cholangiocarcinoma and distal cholangiocarcinoma.

**Table 3:** Relationship between site of stricture, POCS findings and AIP lesion in IgG4-SC patients

Case	Age range^a^ (years)	Sex^a^	IgG4 (mg/dl)	Site of stricture	POCS findings		AIP	Pancreatic enlargement	Enlargement of pancreatic head
					Dilated vessels	Mucosal surface			
1	70–79	1	2590	Extrapancreatic (hilar)	Present	Smooth	Present	Diffuse	Present
2	50–59	1	498	Extrapancreatic (middle)	Present	Smooth	Present	Segmental	Absent
3	50–59	1	408	Extrapancreatic (hilar)	Present	Smooth	Present	Diffuse	Present
4	50–59	1	283	Extrapancreatic (hilar)	Present	Smooth	Present	Diffuse	Present
5	60–69	2	206	Extrapancreatic (hilar)	Present	Smooth	Absent	–	–
6	60–69	1	194	Extrapancreatic (hilar)	Absent	Smooth	Absent	–	–
7	60–69	1	186	Extrapancreatic (hilar)	Present	Smooth	Present	Segmental	Absent
8	60–69	1	21	Extrapancreatic (hilar)	Present	Irregular	Present	Segmental	Absent
9	70–79	1	127	Extrapancreatic (hilar) / intrapancreatic	Present / absent	Smooth / smooth	Present	Focal	Present
10	70–79	1	139	Extrapancreatic (hilar) / intrapancreatic	Present / absent	Irregular / smooth	Present	Focal	Present
11	60–69	1	1620	Intrapancreatic	Present	Smooth	Present	Focal	Present
12	60–69	1	484	Intrapancreatic	Absent	Smooth	Present	Focal	Present
13	50–59	1	426	Intrapancreatic	Absent	Smooth	Present	Diffuse	Present
14	60–69	1	403	Intrapancreatic	Absent	Smooth	Present	Focal	Present
15	70–79	1	264	Intrapancreatic	Absent	Smooth	Present	Focal	Present
16	60–69	1	256	Intrapancreatic	Absent	Smooth	Present	Focal	Present
17	60–69	1	244	Intrapancreatic	Present	Smooth	Present	Diffuse	Present

^a^Age range was used instead of the exact age and sexes were presented by 1 or 2 instead of M or F for securing the patients’ anonymity

*POCS* peroral cholangioscopy; *IgG4-SC* IgG4-related sclerosing cholangitis; *AIP* autoimmune pancreatitis

**Table 4:** Comparison of POCS findings in IgG4-SC patients based on the stricture site

	Extrapancreatic	Intrapancreatic	
	(***n*** = 10)	(***n*** = 9)	***p*** value
Mucosal surface (smooth: irregular)	8: 2	9: 0	0.474
Dilated vessels (present: absent)	9: 1	2: 7	0.006
Tortuosity (present: absent)	7: 2	2: 0	1.000
Abrupt caliber alteration (present: absent)	2: 7	0: 2	1.000
Disruption (present: absent)	1: 8	0: 2	1.000
Easily bleeding (present: absent)	0: 10	0: 9	1.000

*POCS* peroral cholangioscopy; *IgG4-SC* IgG4-related sclerosing cholangitis

A comparison of POCS findings between IgG4-SC and ECC based on the stricture site is shown in Table 5. In comparison between perihilar IgG4-SC and perihilar cholangiocarcinoma, there was a significant difference in terms of the property of mucosal surface and presense of easily bleeding (*P* <  0.001 and 0.003, respectively). There was no significant difference in the frequency of dilated vessels between two groups, but the abrupt caliber alteration and disruption were found significantly more frequently in patients with perihilar cholangiocarcinoma than patients with perihilar IgG4-SC (*P* = 0.002 and 0.041, respectively). In comparison between distal IgG4-SC (intrapancreatic and middle bile duct) and distal cholangiocarcinoma, there was also a significant difference in terms of the property of mucosal surface and presense of easily bleeding (*P* <  0.001 and 0.032, respectively). Furthermore, the frequency of dilated vessels was significantly higher in patients with distal cholangiocarcinoma than patients with dital IgG4-SC (*P* = 0.016).

**Table 5:** Comparison of POCS findings between IgG4-SC and ECC based on the stricture site

	Perihilar bilu duct			Distal bile duct		
POCS findings	IgG4-SC (n = 9)	ECC (***n*** = 30)	***p*** value	IgG4-SC (n = 10^a^)	ECC (***n*** = 23)	***p*** value
Mucosal surface (smooth: irregular)	7: 2	3: 27	< 0.001	10: 0	2: 21	< 0.001
Dilated vessels (present: absent)	8: 1	24: 6	1.000	3: 7	18: 5	0.016
Tortuosity (present: absent)	6: 2	19: 5	1.000	3: 0	16: 2	1.000
Abrupt caliber alteration (present: absent)	2: 6	21: 3	0.002	0: 3	15: 3	0.015
Disruption (present: absent)	1: 7	14: 10	0.041	0: 3	11: 7	0.090
Easily bleeding (present: absent)	0: 9	17: 13	0.003	0: 10	9: 14	0.032

^a^ The 10 strictures consist of 9 intrapancreatic bile ducts and 1 middle bile duct

*POCS* peroral cholangioscopy; *IgG4-SC* IgG4-related sclerosing cholangitis; *ECC* extrahepatic cholangiocarcinoma

### Inter- and intraobserver agreement

The kappa value of interobserver agreement between T.T. and N.S. was 0.719. The kappa value of intraobserver agreement were 0.768 (T.T.) and 0.754 (N.S.).

## Discussion

In this study, the usefulness of POCS in the differential diagnosis between IgG4-SC and ECC was examined. The diagnostic criteria of IgG4-SC [2] has been developed, but its diagnosis remains an important clinical challenge. In particular, patients with IgG4-SC that develop independently without AIP, have normal serum IgG4 levels, or develop during steroid therapy of IgG4-related diseases, are not easy to differentiate from cholangiocarcinoma. Therefore, it is not uncommon for IgG4-SC to be surgically resected on the diagnosis of cholangiocarcinoma [16–20]. Regarding type 1 IgG4-SC, diagnosis is relatively easy because most patients have AIP lesions in the pancreatic head. However, although rare, there has been a report of type 1 IgG4-SC without AIP lesions in the pancreatic head [21], which is not easy to be differentiated from distal cholangiocarcinoma. For the differential diagnosis of benign and malignant bile duct strictures, transpapillary tissue sampling is usually performed during ERCP. Transpapillary tissue sampling methods include aspiration bile cytology, brush cytology, and forceps biopsy. Since the main focus of inflammation of IgG4-SC is under the epithelium of the bile duct, it is not easy to diagnose IgG4-SC by these tissue sampling methods, and the diagnostic outcomes so far reported cannot be considered as good [22–25]. Furthermore, the sensitivity of brush cytology and forceps biopsy in the diagnosis of cholangiocarcinoma have been reported as 20–80% [26–34] and 37–88% [27–30, 32, 33], respectively, and there are large variations by report. In this study, the sensitivity of forceps biopsy in the diagnosis of IgG4-SC was unfortunately 0%, and only 75% of ECC patients had a preoperative pathological diagnosis.

The advantage of POCS is that it allows direct visualization of strictures in detail. In this study, favorable diagnostic performance of POCS (sensitivity 96%, specificity 89%) for distinguishing between ECC and IgG4-SC was obtained by focusing on the property of mucosal surface, the presence of dilated vessels and their morphology, and presence of easily bleeding. On the other hand, dilated vessels were frequently observed in the stricture of perihilar IgG4-SC, so it is essential to carefully observe the morphology of dilated vessels to distinguish them from perihilar cholangiocarcinoma. Another advantage of POCS is that it allows targeted biopsy. In this study, fluoroscopy-guided transpapillary biopsy was selected instead of cholangioscopy-guided biopsy. This is because the video cholangioscopes used in this study did not allow irrigation with saline solution during biopsy and the pathological diagnosis was not easy with small samples obtained with available ultrathin biopsy forceps. Recently, a systematic review [35] demonstrated diagnostic performance of different cholangioscopes in patients with biliary strictures and emphasized the high diagnostic performance of digital single-operator cholangioscope (SpyGlass DS; Boston Scientific Corp.) with improved image quality compared to the fibroptic cholangioscope (SpyGlass; Boston Scientific Corp.). In particular, the advantage of the digital single-operator cholangioscope is that biopsy can be performed under direct visualization while irrigating saline solution, and its sensitivity and specificity in the diagnosis of malignant biliary stricture have been reported to be 68–85% and 98–100%, respectively [36–38]. However, the dual-operator video cholangioscope has better image quality than single-operator digital cholangioscope and is more suitable for evaluating the characteristics of biliary strictures. Two types of endoscope and at least two experienced endoscopists with ERCP-related procedures are required to perform the dual-operator POCS, therefore it cannot easily be performed at any institution. However, the therapeutic strategy of IgG4-SC and cholangiocarcinoma are completely different. Thus, when it is difficult to differentiate IgG4-SC from cholangiocarcinoma even if several diagnostic methods including tissue sampling are performed, the enforcement of dual-operator POCS should be considered.

The characteristic POCS findings at the stricture of IgG4-SC such as smooth mucosal surface, dilated and tortuous vessels without abrupt caliber alteration and disruption, and absence of easily bleeding are considered to reflect its pathological features. The smooth mucosa is considered to reflect the characteristic pathological findings of IgG4-SC in which the main focus of inflammation is under the epithelium of the bile duct, and the epithelium is relatively preserved. Dilated and tortuous vessels have also been reported by Itoi et al. [39] and Yasuda et al. [40], and are considered to be characteristic POCS findings of IgG4-SC. To clarify the difference from the tumor vessels observed in ECC, the morphology of both dilated vessels was compared and evaluated in this study. Tumor vessels are morphologically different from normal vessels and are irregularly shaped, dilated and tortuous, and have blind ends [41]. In gastric cancer [16], colorectal cancer [42], and lung cancer [43], it has been also reported that dilated vessels with caliber alteration, tortuosity, and blind end are useful in diagnosis. The vessels observed at the stricture of IgG4-SC were dilated and tortuous, but abrupt caliber alteration and disruption were significantly less frequent compared to ECC, and the structure of the vascular network was also maintained. These differences in morphology of dilated vessels may be due to the differences in the mechanism of vasodilatation. That is, dilated vessels of cholangiocarcinoma are due to angiogenesis whereas dilated vessels of IgG4-SC may be due to congestion. The easily bleeding was observed to be significantly less frequent in IgG4-SC than in ECC, so it is considered that the wall of the dilated vessel of IgG4-SC is strong as compared to fragile tumor vessels. In addition, in patients with IgG4-SC, improvement in dilatation and tortuosity of vessels immediately after the induction of steroid therapy also supported the congestion. Although the cause of vasodilatation has not been evaluated pathologically, obliterative phlebitis, which is one of the characteristic pathological findings of IgG4-SC, and the stagnation of blood flow due to the spread of inflammation may be the cause.

Regarding the relationship between the stricture site of IgG4-SC and the POCS findings, the mucosal surface was smooth in most patients regardless of the stricture site, but the dilated vessels were found significantly more frequently in the hilar bile duct than in the intrapancreatic bile duct. The characteristic pathological findings of IgG4-SC, such as marked lymphocytic and plasmacytic infiltration, infiltration of IgG4-positive plasma cells, storiform fibrosis, and obliterative phlebitis are not different depending on the site of bile duct [44]. On the other hand, marked spreading of inflammation to peribiliary adipose tissue sometimes forms an inflammatory pseudotumor, which is frequent with IgG4-SC in the hilar bile duct [44, 45]. In addition, since intrapancreatic bile duct stricture is accompanied by an AIP lesion in the pancreatic head in most patients with IgG4-SC, the causes of stricture of the intrapancreatic bile duct may be both IgG4-SC itself and secondary changes due to inflammation and edema of the pancreas [46, 47]. Thus, it is possible that the POCS findings reflect the difference in the degree of spread of inflammation depending on the site and the influences of pancreatic inflammation.

This study found that there were several significant differences between the POCS findings of stricture of IgG4-SC and ECC. However, the evaluation of POCS findings is not well established, lacks objectivity, and the interobserver disagreement can be a significant problem. To the best of our knowledge, this is the first study to examine the reliability of POCS images using a mother-baby scope system. Both the inter- and intraobserver agreement had a kappa value of 0.7 or more, indicating that the reliability of POCS in distinguishing between IgG4-SC and ECC is good.

This study has some limitations. First, it was a retrospective study based on a relatively small number of patients. Second, only one patient with IgG4-SC underwent surgical resection; therefore, histological findings could not be effectively compared with POCS findings for patients with IgG4-SC. The diagnostic criteria [2] and the clinical practice guidelines [48] have been developed; therefore, most IgG4-SC patients can now be definitively diagnosed. Surgical resection will be performed in only a few patients with IgG4-SC; therefore, it is not easy to obtain IgG4-SC histopathological findings that can be compared with POCS findings.

## Conclusions

The characteristic POCS findings of the stricture site of IgG4-SC were smooth mucosal surface, dilated tortuous vessels and lack of easily bleeding. The dilated and tortuous vessels in IgG4-SC had less abrupt caliber alteration and disruption compared to those in ECC, and were frequently found in the extrapancreatic bile duct, especially in the hilar bile duct. Detailed observation of biliary strictures with POCS may help differentiate IgG4-SC from ECC and prevent unnecessary major surgery. In addition, it is necessary to accumulate the number of patients to clarify the POCS findings of IgG4-SC.

## Data Availability

The datasets used and analyzed during the current study are available from the corresponding author on reasonable request.

## Abbreviations

IgG4-SC: IgG4-related sclerosing cholangitis
AIP: Autoimmune pancreatitis
ECC: Extrahepatic cholangiocarcinoma
POCS: Peroral cholangioscopy
ERCP: Endoscopic retrograde cholangiopancreatography
ENBD: Endoscopic naso-biliary drainage
NBI: Narrow band imaging
